# Rapid Classification and Quantitative Prediction of Aflatoxin B_1_ Content and Colony Counts in Nutmeg Based on Electronic Nose

**DOI:** 10.3390/molecules30122538

**Published:** 2025-06-10

**Authors:** Ruiqi Yang, Keyao Zhu, Yuanyu Zhao, Xingyu Guo, Yushi Wang, Jiayu Wang, Huiqin Zou, Yonghong Yan

**Affiliations:** School of Chinese Materia Medica, Beijing University of Chinese Medicine, Beijing 102488, China; yangrq@bucm.edu.cn (R.Y.); 20220935198@bucm.edu.cn (K.Z.); 20220935197@bucm.edu.cn (Y.Z.); 20230941454@bucm.edu.cn (X.G.); 20230935206@bucm.edu.cn (Y.W.); 20230935245@bucm.edu.cn (J.W.)

**Keywords:** nutmeg, mildew, microorganism, electronic nose, AFB_1_, prediction model

## Abstract

The rapid detection and quantification of microbial quantity and aflatoxin are crucial for food safety and quality. In order to achieve rapid detection, nutmeg with mildew, but with difficult-to-observe mildew characteristics, was selected as the research object. Its intrinsic component (dehydrodiisoeugenol) and exogenous noxious substances (the total number of colonies and aflatoxin B_1_) were determined to clarify their changes during the mold process. Subsequently, electronic nose (E-nose) was employed to analyze the odor of nutmeg and was combined with six machine learning algorithms to establish a classification model for samples with different degrees of mold. Finally, three algorithms were chosen as the preferred options to establish the prediction models of indicator content, which can not only identify whether nutmeg is edible but also measure each index. The results demonstrate the enormous potential of E-nose for real-time detection for assessing food safety. In terms of qualitative analysis, the established classification model can achieve a more than 90% true positive rate, suggesting that E-nose could identify early mildew. In quantitative analysis, E-nose combined with Back Propagation Neural Network achieved the highest prediction accuracy, since the correlation coefficient between the predicted value and the measured value of aflatoxin B_1_ is 0.9776, the TAMC is 0.9443, and the TYMC is 0.9685. This study provides a reference for the rapid and comprehensive quality evaluation of mildew-prone nutmeg, and it confirms that E-nose can be applied as a quick and simple technology.

## 1. Introduction

Nutmeg is the seed of *Myristica fragrans* Houtt., which is a medicinal and edible plant. It has been widely used for several centuries around the world, not only as a household spice [1] but also as a herbal medicine for treating gastrointestinal problems such as diarrhea and vomiting. In addition, it is reported that nutmeg has many unique therapeutic effects such as anti-inflammatory, antioxidant, and antifungal activities [2,3,4]. Due to the dual use of nutmeg as a drug and as food, its annual consumption is as high as 140,000 tons. However, it is highly prone to mildew during storage due to its rich volatile components and fatty oil [5], and mildew could reduce or make it lose its efficacy and even produce mycotoxins. Moreover, the mildew of nutmeg occurs from the inside to the outside; when there are visible mildew spots on the nutmeg surface, it has already reached a very high degree of mildew. As a condiment, nutmeg is also commonly sold in the form of powder, and its aromatic nature, which hides the odor produced in the early stage of mildew, makes identifying the presence of mildew difficult.

It is well known that aflatoxins are the most common mycotoxins in food. According to current research, aflatoxin B_1_ (AFB_1_) is the most toxic and most commonly occurring toxin of this group associated with food contamination [6]. On the other hand, even if mycotoxins are not produced, a large number of microorganisms, such as aerobic bacteria and molds, multiply. Microbial limit tests are an important quality control item for food and herbal medicine safety control. The World Health Organization, European Pharmacopeia, United States Pharmacopeia, Japanese Pharmacopeia and Chinese Pharmacopoeia have all developed microbiological limit tests and criteria for herbal medicines and serve as its safety and quality control items. For example, the 2020 edition of the Chinese Pharmacopoeia stipulates that the total aerobic microbial count (TAMC) of decoction pieces for direct oral consumption or steeping in water should not exceed 10^5^ CFU/g, and the total yeast and mold count (TYMC) should not exceed 10^3^ CFU/g [7]. The European Pharmacopoeia generally limits the TAMC for oral herbal medicinal products and extracts to below 10^4^ CFU/g. The United States Pharmacopeia sets a limit of 10^5^ CFU/g for the TAMC and 10^3^ CFU/g for the TYMC for dietary supplements containing herbal medicines [8]. The current method of microbial limit test mainly involves the microbial enumeration test method [8]. Although this method can accurately count the number of microorganisms, it is very time-consuming, taking at least 3 days for the TAMC and 5 days for the TYMC. Meanwhile, current methods for aflatoxin detection and analysis still have notable shortcomings. They are typically expensive, require sophisticated pre-processing, and are non-environmentally friendly. Furthermore, the analytical methods for microorganisms and aflatoxins not only require a range of instruments (e.g., fluorescence detector) and a particular environment, but also skilled operators. In addition, they are non-portable, rendering them unavailable for use in the field.

Therefore, the following questions are raised. Is there a rapid, nondestructive, and robust technique for the detection of microbes and aflatoxins? Is it possible to achieve the rapid and comprehensive evaluation of the quality of food and herbal medicines that are susceptible to mildew and difficult to identify?

The quick detection of microbes and aflatoxins and quality evaluation are extremely essential for food safety and human health. In recent years, there has been increasing interest in developing the electronic nose (E-nose) technique for food and herbal medicine quality control. Fundamentally, E-nose contains a gas sensor array, and the gases emitted by the samples are sensed by the sensor array, which generates feature signal patterns. Due to the advantages of rapidity, high sensitivity, non-invasiveness, and low cost, E-nose has been widely used in many fields such as the quality evaluation of herbal medicines [9], environmental monitoring [10], food adulteration and safety [11,12], and medical diagnosis [13]. Furthermore, E-nose can be applied not only for qualitative analysis but also for quantitative analysis. By sensing the odor composition and concentration of the tested substance, and with the help of intelligent algorithms, the E-nose system can perform qualitative and semiquantitative detection. For example, E-nose has been combined with artificial neural network (ANN) modeling for the qualitative and quantitative analyses of benzoic acid in cola-type beverages [14]. E-nose has been coupled with support vector machine regressors to detect varying adulteration levels in camellia oil [15]. In addition, E-nose has been already applied to study bacterial discrimination, prediction, and classification [16,17], even for certain pathogenic bacteria [18,19]. Moreover, it has been confirmed that E-nose supported by an ANN could be a rapid and reliable tool for the detection of AFB_1_ and fumonisins in maize [20].

In this study, nutmeg samples with different degrees of mildew were obtained with accelerated experiment and natural reserved tests and were tested for dehydrodiisoeugenol (the main active ingredient of nutmeg) [21,22] and AFB_1_ via HPLC, TAMC, and TYMC via microbial enumeration tests, and the odor information was acquired via E-nose, which consisted of 12 metal oxide gas sensors. Ultimately, the classification model of nutmeg was built to evaluate the presence or absence of mildew, and the content prediction models were established by combining three machine learning methods to achieve accurate prediction. Based on the above models, the rapid and comprehensive quality and safety evaluation of food and herbal medicines was achieved.

## 2. Results and Discussion

### 2.1. Changes in Physicochemical Indexes During Storage of Nutmeg

**Figure 1:**
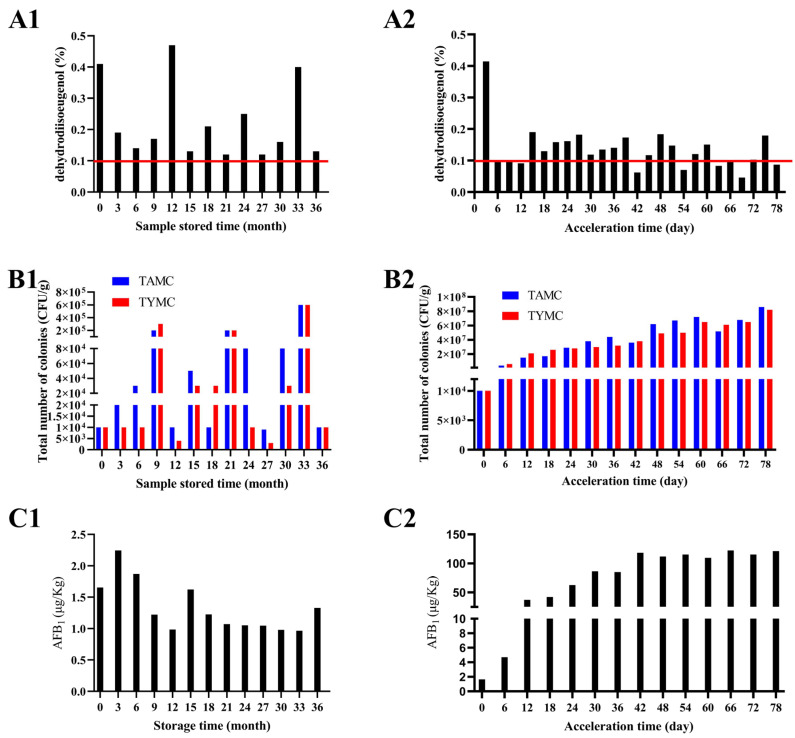
Changes in dehydrodiisoeugenol content, total number of colonies, and AFB_1_ content during storage of nutmeg (**A1**) dehydrodiisoeugenol of natural reserved group; (**A2**) dehydrodiisoeugenol of accelerated group; (**B1**) total number of colonies of natural reserved group; (**B2**) total number of colonies of accelerated group; (**C1**) AFB_1_ of natural reserved group; (**C2**) AFB_1_ of accelerated group.

The dehydrodiisoeugenol, total number of colonies, and AFB_1_ content of nutmeg samples during different storage times are presented in Figure 1. As shown in Figure 1(A1,A2), the red horizontal line is the content limit of dehydrodiisoeugenol from the Chinese Pharmacopoeia for nutmeg, which states that the content of dehydrodiisoeugenol should not be less than 0.1%. All the natural reserved samples comply with the standard, and the high values of dehydrodiisoeugenol observed at 12, 24, and 33 months may be attributed to the conversion of other components into dehydrodiisoeugenol over an extended storage time. Another contributing factor could be the individual variations inherent to nutmeg itself [23], which require further investigation. However, the content of dehydrodiisoeugenol in the accelerated sample dropped below the standard from the sixth day of acceleration. With the extension of the acceleration period, the content of dehydrodiisoeugenol in the samples fluctuated at the standard line.

**Figure 2:**
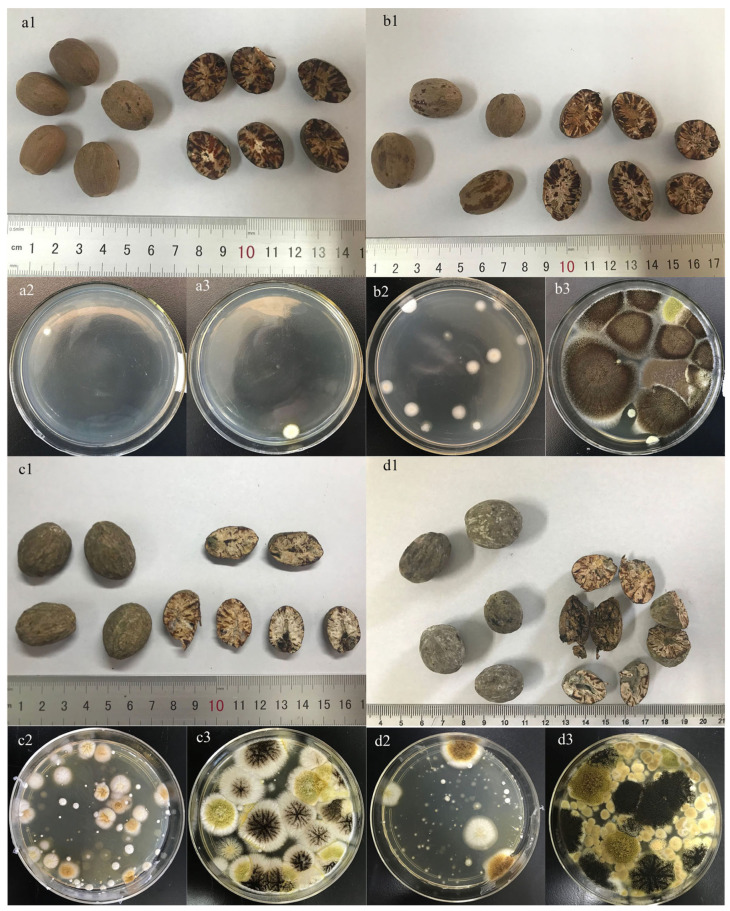
Nutmeg samples and TAMC and TYMC ((**a1**) L1 sample; (**a2**) L1 sample of TAMC; (**a3**) L1 sample of TYMC; (**b1**) J2 sample; (**b2**) J2 sample of TAMC; (**b3**) J2 sample of TYMC; (**c1**) J7 sample; (**c2**) J7 sample of TAMC; (**c3**) J7 sample of TYMC; (**d1**) J13 sample; (**d2**) J13 sample of TAMC; (**d3**) J13 sample of TYMC).

The TAMC and TYMC test results are shown in Figure 1(B1,B2). The total number of colonies increased with the acceleration time in the accelerated group, while in natural reserved group, it rose in September and decreased in December as the humidity in Beijing (China) is relatively high in September (July to September is the rainy season in Beijing, and the environmental humidity is about 75%). The optimal growth temperatures for aerobic bacteria, molds, and yeasts generally range from 20 °C to 30 °C, with a humidity level of 60–85%. From January to September, as temperatures rise and air humidity increases (particularly during the plum rain season in summer), ideal breeding conditions are created for microorganisms, leading to the rapid accumulation of microbial populations on or within medicinal herbs. In winter, with the decrease in temperature (Beijing, China) and drop in air humidity, the metabolic rates of microorganisms slow down, spores enter a dormant state, and there is a significant reduction in colony formation. Although the total number of colonies were decreased with the decrease in humidity, the produced fungal toxins did not similarly decrease. As the mold intensified, not only did the number of colonies increase, but the morphology of the colonies also became more and more diverse. Figure 2a is the mildew-free sample, which has an obvious marble-like pattern and gives the cut surface of nutmeg an oily appearance. Figure 2b is a moldy sample. Although no obvious hyphae or moldy spots were produced at this time, the marble-like pattern on the cut surface of nutmeg was not obvious, and a characteristic smell was produced (which was difficult to detect for humans, but could be captured by E-nose). Meanwhile, the number of colonies increased significantly at this time. Figure 2c,d is a highly moldy sample, and it is evident that the nutmeg cut surface has obvious moldy hyphae; the morphology and color of the colony have greatly changed, which indicate that the bacterial flora has become diverse.

In the microbial limit experiment, although the total number of colonies decreased as the humidity decreased, the toxins already produced did not decrease accordingly. Therefore, comprehensive evaluation is required in food and herbal medicine evaluation, rather than relying only on a single index, which shows the advantage of E-nose. The best time to control mildew is before the exponential phase of mold growth, but this method has a long detection cycle and does not facilitate the use of quick measures for controlling the occurrence of mold. Thus, it is necessary to combine the volatile substances in the process of mildew for the early warning of mold [24]. Fortunately, E-nose can achieve the detection of volatile substances.

The AFB_1_ content in the natural reserved sample group is low and does not exceed the standard limit recommended by the Chinese Pharmacopoeia (5 μg/kg). However, it reaches 37.24 μg/kg after 12 days of acceleration in the accelerated group, far exceeding the standard limit (Figure 1(C1,C2)). Based on the AFB_1_ content, the samples were categorized into three classes (Table 1): (1) the normal group (green, AFB_1_ < 5 μg/kg, below the standard limit); (2) the moldy group (orange, 5 μg/kg < AFB_1_ < 100 μg/kg); and (3) the highly moldy group (red, AFB_1_ > 100 μg/kg). Among the 26 batches of the analyzed nutmeg samples, 14 batches were normal (group 1), 5 batches were moldy (group 2), and 7 batches were highly moldy (group 3).

**Figure 3:**
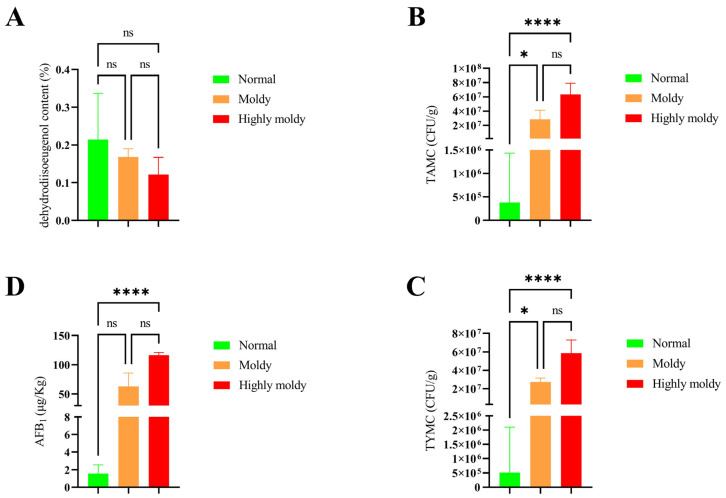
Analysis of dehydrodiisoeugenol, TAMC, TYMC, and AFB_1_ of nutmeg samples with different degrees of mildew using the Kruskal–Wallis test ((**A**) dehydrodiisoeugenol; (**B**) TAMC; (**C**) TYMC; (**D**) AFB_1_. * represents *p *< 0.05; **** represents *p *< 0.0001, ns represents *p* > 0.05).

Analysis was performed using the Kruskal–Wallis test for assessing the dehydrodiisoeugenol content, along with the TAMC, TYMC, and AFB_1_ content of nutmeg samples with different degrees of mildew; the results are shown in Figure 3. This illustrates that the dehydrodiisoeugenol content is not significantly different in the three groups (*p* > 0.05), while the TAMC, TYMC, and AFB_1_ content are significantly different in the three groups (*p* < 0.0001). During the experiment, it was observed that the nutmeg samples exposed to mold showed a decrease in their active component content. In addition, mold facilitates the growth of a significant number of microorganisms and generates mycotoxins. Unfortunately, nutmeg mold is not easily detected since it occurs from the inside to the outside. Moreover, in many cases, nutmeg is consumed in the form of powder, making the mold even less noticeable, thus posing a potential threat to human health. Therefore, it is necessary to combine other methods to evaluate the safety and quality of nutmeg.

**Figure 4:**
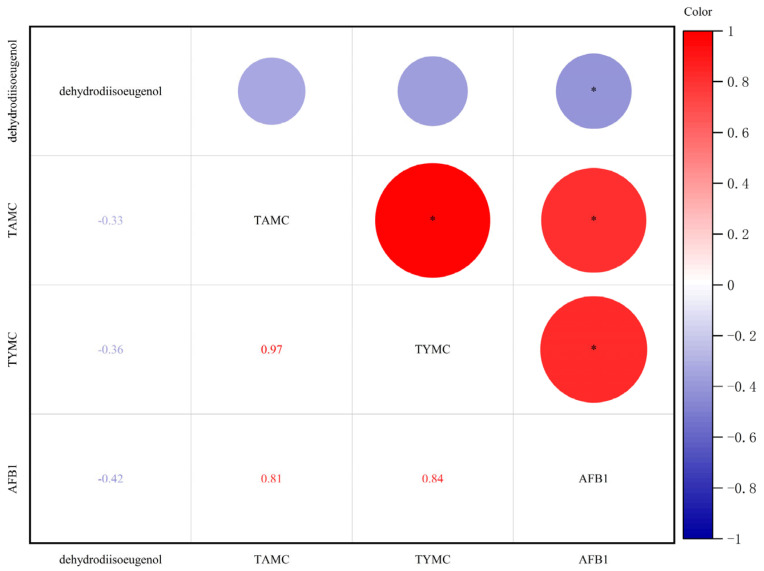
Spearman’s correlation analysis based on dehydrodiisoeugenol, TAMC, TYMC, and AFB_1_. Color of circle denotes nature of correlation, with 1 indicating perfect positive correlation (dark red) and −1 indicating perfect negative correlation (dark blue). Strong correlations and weak correlations are indicated by darker-colored circles and lighter-colored circles, respectively. * represents *p* < 0.05.

It is evident from Figure 4 that there is a significant negative correlation between the AFB_1_ content and the dehydrodiisoeugenol content, indicating that as the proportion of AFB_1_ increases, the content of the active ingredient dehydrodiisoeugenol decreases. At the same time, there is a significant positive correlation between the AFB_1_ content and the TAMC and the TYMC, and the growth of colonies is accompanied by the production of aflatoxin.

### 2.2. Odor Characteristics

**Figure 5:**
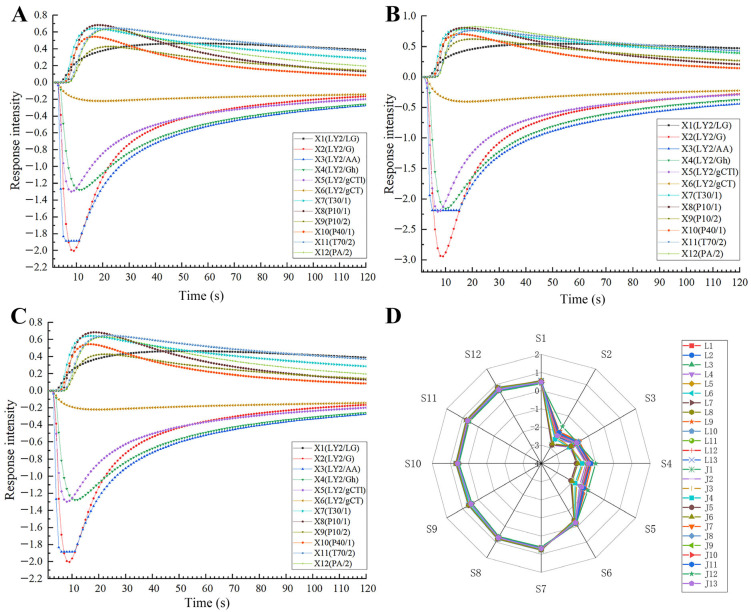
Response curve of 12 E-nose sensors and radar map ((**A**) normal; (**B**) moldy; (**C**) highly moldy; (**D**) radar map).

The response curve of the 12 E-nose sensors for the nutmeg samples with different degrees of mildew are shown in Figure 5A–C. The intensities of E-nose sensor responses increased and reached their peak value by approximately 15 s. Subsequently, the response value slowly decreased and finally gradually reached a steady state at 60 s. A radar map was employed to visualize the changes in the 12 sensors, as shown in Figure 5D. It is evident from Figure 5 that the absolute value of the maximum response value of sensors S2 and S3 is higher, followed by sensors S4 and S5, and the minimum response value of sensor S6 is lower. The most significant changes in response values were observed for the sensors S2, S3, S4, and S5, while the other response values of sensors were almost identical and overlapped. These four sensors, S2, S3, S4, and S5, that showed significant changes were sensitive to ammonia/organic amine, carbon monoxide, ethanol, and hydrogen sulfide, respectively, suggesting that these kinds of chemicals may be produced or altered in moldy nutmeg.

### 2.3. Qualitative Classification of Mold Degree of Nutmeg

In this study, six classification algorithms were used to assess the effectiveness of E-nose in distinguishing different mold samples. The commonly used evaluation metrics used for classification include Kappa statistic, accuracy, recall, precision, F-measure, true positive rate (TPR), false positive rate (FPR), true negative rate (TNR), false negative rate (FNR), etc.

Kappa statistics are a measure of agreement normalized for chance agreement. It is positively correlated with both the area under the curve (AUC) and accuracy. Thus, the closer the Kappa statistic is to 1, the better is the performance of the model. Recall is used to measure the model’s ability to correctly predict all actual positive samples. A high recall value indicates that the model has efficiency of finding positive samples. Precision denotes the proportion of samples classified in the positive categories that are truly positive. F-measure is used to comprehensively evaluate the performance of the classification algorithm; the closer the value of F-measure is to 1, the better is the performance of the classification algorithm [25].

A total of 26 batches of nutmeg samples, with 7 replicate measurements for each batch, totaling 182 sets of data, were used for establishing the models. The maximum response values from the 12 E-nose sensors were used as the inputs. We selected common metrics from two dimensions: model validity and data categorization. The overall categorization results for ten-fold cross-validation and external test validation (70%) are shown in Table 2 (the partitioning of the dataset was automatically performed using the software Weka 3.9.0). The TPR of all classification models is higher than 90% except for that of SMO. By comparing the classification results of ten-fold cross-validation and external test validation, it is found that the ten-fold cross-validation results of the models are overall higher than the external test validation results. In terms of “function”, the BPNN model has better classification ability. Both IBK and Kstar from Lazy has the best classification ability, and IBK slightly outperforms Kstar. The classification models based on the decision tree algorithm are second only to those based on the lazy algorithm, while the Random Forest prediction performs better.

Among all six algorithms, the IBK classification model combined with ten-fold cross-validation performed the best with a Kappa statistic score of 0.9908, a precision of 0.995, a recall of 0.995, and an F-measure score of 0.994. The results demonstrate that a classification model built using E-nose combined with the IBK algorithm could effectively identify the different degrees of mold contamination of nutmeg.

### 2.4. Quantitative Prediction of Physicochemical Indexes of Nutmeg

According to Section 2.3 three algorithms, i.e., BPNN, IBK, and Random Forest, were selected to build the prediction models for the dehydrodiisoeugenol content, TAMC, TYMC, and AFB_1_ content. For each method, the external testing set validation and ten-fold cross-validation were performed for determining the assessment capability of E-nose (Table 3).

**Figure 6:**
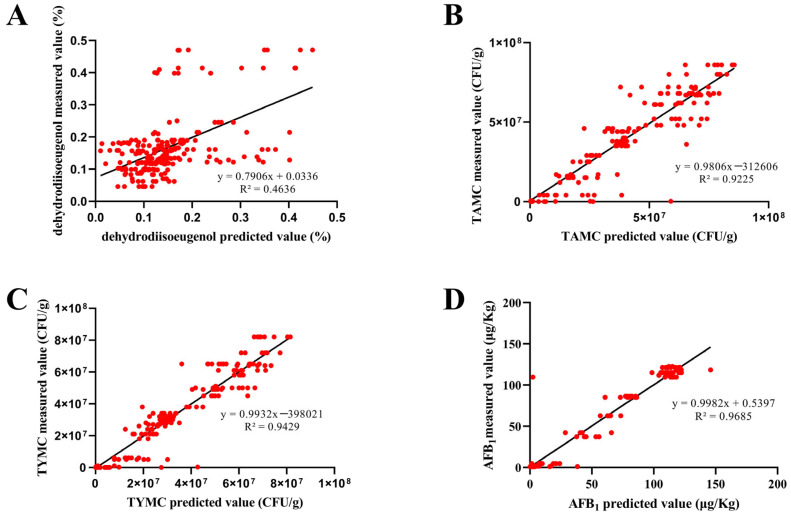
Scatter plot of measured and predicted values based on BPNN with external testing set validation (**A**) dehydrodiisoeugenol; (**B**) TAMC; (**C**) TYMC; (**D**) AFB_1_.

Among the prediction models of the four indicators, BPNN performed the best, with a significant correlation between the predicted values and the measured values combined with external testing set validation; the CC was 0.4856 for the dehydrodiisoeugenol content, 0.9443 for the TAMC, 0.9685 for the TYMC, and 0.9776 for the AFB_1_ content. And meanwhile, the RAE were also low. The dehydrodiisoeugenol prediction model performed poorly, and the correlation coefficient between the predicted values and the measured values displayed by all three classifiers was less than 0.6. Based on the analysis of the results of dehydrodiisoeugenol determination, it was inferred that mildew has little impact on the content of dehydrodiisoeugenol. In other words, the dehydrodiisoeugenol content did not change significantly with the worsening of mildew, which may lead to the poor performance of the prediction model. The intuitive scatter plot of the measured and predicted values is shown in Figure 6. It is evident from Figure 6 that all R^2^ values for the predicted and measured values of TAMC, TYMC, and AFB_1_, based on the BPNN model, were higher than 0.9 (except dehydrodiisoeugenol). The results presented above indicate that E-nose combined with machine learning could achieve a high degree of accuracy in forecasting, providing a reference for the rapid detection of food and herbal medicines.

Although E-nose combined with the BPNN models demonstrates certain application potential in predicting the degree of mildew, TAMC, TYMC, and AFB_1_ content, the method still has some limitations. The sensor responses of E-noses are significantly affected by environmental factors (such as temperature and humidity) and sample conditions (such as matrix interference), which may lead to data fluctuations or noise [26]. If the training data fail to cover all possible interfering factors, the predictive accuracy of the model in complex real-world scenarios declines. Additionally, the decision-making process of the BPNN model, a nonlinear model, lacks transparency, making it difficult to elucidate the specific correlations between sensor responses and target variables. This limitation may arise in scenarios where clear causal relationships are required. To address these issues, approaches like sensor calibration, outlier removal, and feature selection could be integrated to optimize data preprocessing, thereby enhancing data quality. Furthermore, SHapley Additive exPlanations (SHAP) or Local Interpretable Model Agnostic Explanation (LIME) [27] could be employed to dissect the decision logic of BPNN, strengthen model interpretability, and improve the credibility of the results.

## 3. Materials and Methods

### 3.1. Chemical Reagents and Materials

Trypticase soy agar medium (TSA) and Sabouraud dextrose agar (SDA) were procured from Beijing SanYao Science Technology Development Co (Beijing, China). Dehydrodiisoeugenol, the reference substance (lot 3270), was purchased from Shanghai Nature-standard Technical Service Co., Ltd. (Shanghai, China). AFB_1_, the reference substance, and immunoaffinity columns were purchased from Beijing Clovertech Limited Co. (Beijing, China). Methanol and acetonitrile were HPLC-grade; all other chemicals were of analytical grade.

### 3.2. Samples Collection

Nutmeg samples were purchased from Hebei Xinghua Traditional Chinese Medicine Co., Ltd. ( Heibei, China) and were authenticated based on macroscopical identification by Professor Yong-Hong Yan from Department of Chinese Materia Medica of Beijing University of Chinese Medicine. Samples with different degrees of mildew were prepared using the accelerated experiment and the natural reserved test. In the natural reserved test, the nutmeg samples were wrapped in plastic bags (made of polyethylene) and stored indoors (Beijing, China) protected from light for three years, with samples taken every three months. Batch 2017.01 (bought nutmeg that was harvested in 2017) was not sampled in the first year and was sampled in March 2018, i.e., 15-month sample. Batch 2016.01 (bought nutmeg that was harvested in 2016) was not sampled in the first two years and was sampled in March 2018, i.e., 27-month sample. The L1 sample from batch 2018.1 was used for accelerated testing, and the unpacked samples were laid flat on a tray and stored at a temperature of 30 °C and a humidity of 95% RH (nutmeg is susceptible to aflatoxin growth at this temperature and humidity [28]). Nutmeg samples were taken every 6 days, and 13 batches of samples were obtained with accelerated testing for 78 days, which were numbered from J1 to J13. The samples’ information is shown in Table 4. A total of 2.5 kg of samples was used for the natural reserved test for each batch, and 7 kg of samples was used for accelerated testing, with 500 g taken at each sampling occasion for various tests. For each test, at least six seeds were selected for grinding to ensure biological replication. All samples were tested for microbiological counts immediately after sampling and subsequently stored at −20 °C and tested for other indicators, such as dehydrodiisoeugenol, after all samples were tested.

### 3.3. Physicochemical Identification

#### 3.3.1. Determination of Dehydrodiisoeugenol Content

The content of dehydrodiisoeugenol was determined according to the Chinese Pharmacopoeia. 

Sample preparation: A 0.5 g quantity of nutmeg powder was accurately weighed in a conical flask. A 50 mL volume of methanol was precisely added, and the total weight of the mixture was determined. The mixture was sonicated for 30 min (power: 250 W; frequency: 40 KHz). After cooling, it was weighed again, and the lost weight was made up with methanol. The mixture was then shaken well and filtered, and the subsequent filtrate was collected.

HPLC: The HPLC equipment was Waters e2695-2489 (Waters Corporation, Milford, MA, USA). The chromatographic column used was ZORBAX SB-C18 (4.6 × 250 mm, 5 μm) (Agilent Technologies, Santa Clara, CA, USA). The other parameters used for the HPLC process are as follows: mobile phase: methanol/water (75:25); velocity of flow: 1.0 mL/min; injection volume: 10 μL; and detection wavelength: 274 nm.

#### 3.3.2. Microbial Counting Protocols

Microbial counting protocols were established and validated by referring to the 2015 edition of the Chinese Pharmacopeia general rule 1105 microbial limit test for non-sterile products: microbial enumeration test [29]. Preparation of test solution: The nutmeg slices were placed in a grinder wiped with an alcohol cotton ball, they were crushed, and the nutmeg powder was transferred to a sterilized bag with a sterilized spoon. A 10 g quantity of the nutmeg powder was taken in a sterilized mortar, and 100 mL of sterile physiological saline containing 0.1 mL of polysorbate 80 was added to the mortar; the mixture was quickly ground till dissolution to make a 1:10 test solution. The 1:100, 1:1000, 1:10,000, 1:100,000, and 1:1,000,000 diluents were prepared in sequence. Determination of TAMC: a 1 ml volume of gradient diluent was transferred to TSA, and it was inverted in a constant-temperature incubator at 30–35 °C for 48 h. Determination of TYMC: a 1 ml volume of gradient diluent was transferred to SDA, and it was inverted in a constant-temperature incubator at 20–25 °C for 72 h.

#### 3.3.3. Determination of AFB_1_ Content

The AFB_1_ content was determined using the HPLC combined fluorescence detector. The HPLC equipment was Shimadzu LC-20A, Tokyo, Japan. The chromatographic column was Cloversil-C18 (4.6 × 250 mm, 5 μm) ( Wuhan Ecalbio, Wuhan, China). The other parameters used for the HPLC process are as follows: mobile phase: methanol/water (45:55); velocity of flow: 0.8 ml/min; and injection volume: 20 μL. The fluorescence detector excitation and emission wavelengths were set at 360 nm and 440 nm, respectively.

Sample preparation: A 5.0 g quantity of nutmeg powder was accurately weighed in a 50 mL centrifuge tube. A 25 mL volume of an aqueous solution containing 80% acetonitrile was accurately measured and added to the centrifuge tube. The mixture was shaken on a shaker for 30 min. It was then filtered through microfiber filter paper, and 1.0 mL of the filtrate was accurately transferred. A 9.0 mL volume of 5% Tween-20 PBS7.0 solution was added to dilute the filtrate. A 10.0 mL volume of the sample diluent was accurately transferred into a glass syringe. The air pressure pump was connected to the glass syringe, and the pressure was adjusted to allow the solution to slowly pass through the immunoaffinity column at a flow rate of approximately 2 mL/min until 2~3 mL of air passed through the column. The column was rinsed twice with 10.0 mL of water at a flow rate of 2–3 mL/min, and the entire effluent volume was discarded, and 2~3 mL of air was allowed to pass through the column. A 1.0 mL volume of chromatography-grade methanol was accurately transferred to elute the column, and the entire volume of eluate was collected in a 2 mL volumetric flask. It was diluted to the mark with pure water to obtain the final solution. The PBS solution was prepared by accurately weighing 8.00 g of NaCl, 1.44 g of Na_2_HPO_4_, 0.24 g of KH_2_PO_4_, and 0.20 g of KCl, which were then dissolved in 990 mL of pure water. The pH of the solution was adjusted to 7.0 with hydrochloric acid; finally, the solution was diluted to 1000 mL with pure water.

The above three detection methods (Section 3.3.1, Section 3.3.2 and Section 3.3.3) all used nutmeg powder (passed through a 24-mesh sieve), and the same sample was assessed in parallel three times.

### 3.4. E-Nose Analysis

α-Fox3000 E-nose (Alpha MOS, Co., Ltd., Toulouse, France), with 12 metal oxide gas sensors, was employed to obtain the odor information of nutmeg; the names and response characteristics of each sensor are presented in Table 5. E-nose was self-checked, and the sensor array was preheated for 2–3 h before each sampling experiment. Processed pure air was used as the carrier gas to clean the sensor array, and it restored the signal response back to the baseline level. Nutmeg powder sample (0.2 g) was sealed into a 10 mL vial and incubated using an E-nose autosampler for 60 s at 35 °C (at 250 rpm). The temperature and volume of injection were set at 45 °C and 300 μL. The flow rate of carrier gas was 150 mL/min. The data acquisition interval and data acquisition cycle were 1 s and 120 s, respectively. Every nutmeg sample was continuously sampled 7 times (separate vials), and the maximum response values of E-nose sensor were obtained and used for further analysis.

### 3.5. Data Analysis

#### 3.5.1. Physicochemical Analysis

The trends of dehydrodiisoeugenol content, AFB_1_ content, and the total number of colonies were visualized using bar charts. Based on the measured AFB_1_ levels, the samples were classified into three mold groups. These groups were designated as one to three based on the severity of mold contamination and the potential health risks associated with each sample. The differences between groups with different mold grades were analyzed using the Kruskal–Wallis test, with the significant difference set at *p* < 0.05 by Duncan’s multiple tests. These analysis and abovementioned images were implemented through the software GraphPad Prism (version 9.4.0). Spearman’s correlation analysis for the various physicochemical indexes was performed using the software Origin 2022.

#### 3.5.2. Classification Analysis

When the food and herbal medicines have mildew, they produce specific odors such as acid gas, distiller’s grain gas, and moldy gas. The main components resulting in these odors are hydroxyl, aldehyde, sulfide, and other compounds that are produced by microorganisms, resulting in a certain systematic difference between the volatile odor characteristics of the samples without mildew and their odor characteristics in the early stage of mildewing, which is the basis on which the E-nose gas sensor has been developed. Studies have shown that, during the storage of nutmeg, the production of volatile organic compounds such as ammonia/organic amines, carbon monoxide, ethanol, and hydrogen sulfide, as well as changes in the terpene and phenylpropene components of nutmeg, may be the reason for odor changes [30].

In this study, the maximum response values of all 12 sensors were chosen for analysis. Initially, a radar map was generated depicting the response values of E-nose, aiming to identify which sensor types exhibit variability during the mold process. Moreover, the classification models of nutmeg with different degrees of mildew based on E-nose were established using six machine learning algorithms, i.e., Back Propagation Neural Network (BPNN), Sequential Minimal Optimization (SMO), Instance-based Learning (IBK), Kstar, Random Forest (RF), and Random Tree (RT), with the software Weka. The external test set validation and ten-fold cross-validation method were used to evaluate the performance of all six classification models.

The machine learning algorithms BPNN and SMO were able to “function” in the software Weka. SMO implements John Platt’s Sequential Minimal Optimization Algorithm for training a support vector classifier. While BPNN is a classifier that uses back propagation to learn a multi-layer perceptron to classify instances. IBK and Kstar were attributed to “lazy” in Weka, and they are both instance-based algorithms. The difference is that IBK is based on a K-nearest neighbors classifier, while Kstar is an entropy-based distance function [31]. Random Forest and Random Tree belongs to “trees” in Weka, which is well known for its decision tree-based algorithms. Random Forest is class for constructing a forest of random trees, and the term “forest” refers to a series of decision trees [32]. Random Tree is used for constructing a tree that considers K randomly chosen attributes at each node.

#### 3.5.3. Prediction Models 

Combining the classification results of the above six machine learning algorithms, the algorithm with the highest accuracy was selected to build the prediction models for the dehydrodiisoeugenol content, AFB_1_ content, TAMC, and TYMC. The prediction abilities of predictive models were validated using the ten-fold cross-validation and external test sets. The validation and comparison of these models were performed using different quantitative indexes, such as correlation coefficient (CC) and relative absolute error (RAE). The modeling and data process were designed using Weka 3.9.0.

## 4. Conclusions

This study demonstrates the enormous potential of E-nose for real-time detection in assessing food safety. The study results showed that E-nose combined with machine learning methods can be used for the qualitative and quantitative analyses of mildew in nutmeg. This method could not only evaluate the edibility/inedibility of nutmeg, but also could assess the total number of colonies and the aflatoxin content, providing a basis for the rapid and comprehensive detection of this kind of mildew-prone food and herbal medicines.

In recent years, E-nose techniques have been explored for the detection of microbes, particularly pathogenic bacteria. In subsequent research, we will continue to isolate and identify pathogenic bacteria and determine other mycotoxins to establish prediction models using E-nose for the rapid and comprehensive quality evaluation of food and herb medicines. In addition, we will continue to conduct diverse experiments, increase the number of samples, and optimize the prediction model to further improve its prediction accuracy.

In summary, E-nose is an ideal methodology for online process control, providing simple and rapid qualitative and quantitative detection, and it requires little or no sample preparation and reagent consumption. However, most of the E-nose techniques currently used in laboratories are not miniaturized. We hope that these sensors will be miniaturized into small devices in the future, making them portable and providing promising detection tools for everyday use.

## Figures and Tables

**Table 1:** AFB_1_ contamination level of samples.

Natural Retention Samples			Accelerated Test Samples		
Sample Number	AFB_1_ (μg/kg)	Mold Degree	Sample Number	AFB_1_ (μg/kg)	Mold Degree
L1	1.6530	Class 1	J1	4.7052	Class 1
L2	2.2458	Class 1	J2	37.2449	Class 2
L3	1.8692	Class 1	J3	42.2726	Class 2
L4	1.2226	Class 1	J4	62.6829	Class 2
L5	0.9841	Class 1	J5	86.3746	Class 2
L6	1.6227	Class 1	J6	85.0230	Class 2
L7	1.2259	Class 1	J7	118.3960	Class 3
L8	1.0726	Class 1	J8	111.7690	Class 3
L9	1.0500	Class 1	J9	115.0944	Class 3
L10	1.0461	Class 1	J10	109.6292	Class 3
L11	0.9798	Class 1	J11	122.3826	Class 3
L12	0.9661	Class 1	J12	115.0990	Class 3
L13	1.3292	Class 1	J13	121.3189	Class 3

**Table 2:** Evaluation of classification models.

	Classification	External Testing Set Validation					Ten-Fold Cross-Validation				
		Kappa Statistic	TPR	Precision	Recall	F-Measure	Kappa statistic	TPR	Precision	Recall	F-Measure
Function	BPNN	0.8971	0.935	0.95	0.935	0.937	0.9544	0.973	0.973	0.973	0.973
	SMO	0.6415	0.79	0.824	0.79	0.771	0.6111	0.78	0.825	0.78	0.749
Lazy	IBK	0.9469	0.968	0.969	0.968	0.968	0.9908	0.995	0.995	0.995	0.994
	Kstar	0.921	0.952	0.955	0.952	0.952	0.9908	0.995	0.995	0.995	0.994
Trees	RF	0.8955	0.935	0.943	0.935	0.937	0.9544	0.973	0.973	0.973	0.973
	RT	0.8938	0.935	0.935	0.935	0.935	0.8637	0.918	0.919	0.918	0.918

**Table 3:** Evaluation values of three predictive models.

	Classification	External Testing Set Validation		Ten-Fold Cross- Validation	
		CC	RAE	CC	RAE
Dehydrodiisoeugenol	BPNN	0.4856	149.1%	0.3734	111.9%
	IBK	0.5595	80.08%	0.4212	79.67%
	RF	0.4646	91.04%	0.5061	82.14%
TAMC	BPNN	0.9443	27.82%	0.934	27.60%
	IBK	0.9344	10.96%	0.9671	7.90%
	RF	0.9010	27.60%	0.9184	23.26%
TYMC	BPNN	0.9685	23.69%	0.9598	21.83%
	IBK	0.9214	13.31%	0.9571	9.83%
	RF	0.8985	27.66%	0.9035	25.36%
AFB_1_	BPNN	0.9776	15.83%	0.9792	15.41%
	IBK	0.9552	6.76%	0.9861	3.09%
	RF	0.9395	22.96%	0.9340	20.87%

**Table 4:** Samples’ information.

Natural Retention Samples						Accelerated Test Samples		
Sample Batch	Numbers	Storage Time (Month)	Sampling Time	Temperature (°C)	Humidity (%)	Sample Batch	Numbers	Acceleration Time (Day)
2018.01	L1	0	2018.01	15	40	2018.01	L1	0
2018.01	L2	3	2018.03	18	42	2018.01	J1	6
2018.01	L3	6	2018.06	26	50	2018.01	J2	12
2018.01	L4	9	2018.09	25	60	2018.01	J3	18
2018.01	L5	12	2018.12	16	38	2018.01	J4	24
2017.01	L6	15	2018.03	18	42	2018.01	J5	30
2017.01	L7	18	2018.06	26	50	2018.01	J6	36
2017.01	L8	21	2018.09	25	60	2018.01	J7	42
2017.01	L9	24	2018.12	16	38	2018.01	J8	48
2016.01	L10	27	2018.03	18	42	2018.01	J9	54
2016.01	L11	30	2018.06	26	50	2018.01	J10	60
2016.01	L12	33	2018.09	25	60	2018.01	J11	66
2016.01	L13	36	2018.12	16	38	2018.01	J12	72
						2018.01	J13	78

**Table 5:** Detailed information of 12 metal oxide sensors (α-Fox3000 E-nose, Alpha MOS).

No.	Type of Sensor	Sensitive Substance
S1	LY2/LG	Oxidizing gas
S2	LY2/G	Ammonia, carbon monoxide
S3	LY2/AA	Ethanol
S4	LY2/GH	Ammonia/organic amine
S5	LY2/gCTL	Hydrogen sulfide
S6	LY2/gCT	Propane/butane
S7	T30/1	Organic solvents
S8	P10/1	Hydrocarbons
S9	P10/2	Methane
S10	P40/1	Fluorine
S11	T70/2	Aromatic compounds
S12	PA/2	Ethanol, ammonia/organic amine

## Data Availability

The data are contained within the article.

## Abbreviations

The following abbreviations are used in this manuscript:

AFB_1_	Aflatoxin B_1_
TAMC	Total aerobic microbial count
TYMC	Total yeast and mold count
E-nose	Electronic nose
BPNN	Back Propagation Neural Network
SMO	Sequential Minimal Optimization
IBK	Instance-based Learning
RF	Random Forest
RT	Random Tree
CC	Correlation coefficient
RAE	Relative absolute error
